# Enhancing the fatty acid profile of milk through forage‐based rations, with nutrition modeling of diet outcomes

**DOI:** 10.1002/fsn3.610

**Published:** 2018-02-28

**Authors:** Charles M. Benbrook, Donald R. Davis, Bradley J. Heins, Maged A. Latif, Carlo Leifert, Logan Peterman, Gillian Butler, Ole Faergeman, Silvia Abel‐Caines, Marcin Baranski

**Affiliations:** ^1^ Bloomberg School of Public Health Johns Hopkins University Baltimore MD USA; ^2^ Benbrook Consulting Services Troy OR USA; ^3^ Biochemical Institute University of Texas at Austin Austin TX USA; ^4^ West Central Research and Outreach Center University of Minnesota Morris MN USA; ^5^ Organic Valley/CROPP Cooperative Lafarge WI USA; ^6^ Centre for Organics Research Southern Cross University Lismore NSW Australia; ^7^ School of Natural and Environmental Science Newcastle University Newcastle upon Tyne UK; ^8^ Department of Cardiology Aarhus University Hospital Aarhus Denmark

**Keywords:** dairy farming, dairy fatty acids, grass milk, Grassmilk^TM^, omega‐6/omega‐3 ratio, organic milk

## Abstract

Consumer demand for milk and meat from grass‐fed cattle is growing, driven mostly by perceived health benefits and concerns about animal welfare. In a U. S.‐wide study of 1,163 milk samples collected over 3 years, we quantified the fatty acid profile in milk from cows fed a nearly 100% forage‐based diet (grassmilk) and compared it to profiles from a similar nationwide study of milk from cows under conventional and organic management. We also explored how much the observed differences might help reverse the large changes in fatty acid intakes that have occurred in the United States over the last century. Key features of the fatty acid profile of milk fat include its omega‐6/omega‐3 ratio (lower is desirable), and amounts of total omega‐3, conjugated linoleic acid, and long‐chain omega‐3 polyunsaturated fatty acids. For each, we find that grassmilk is markedly different than both organic and conventional milk. The omega‐6/omega‐3 ratios were, respectively, 0.95, 2.28, and 5.77 in grassmilk, organic, and conventional milk; total omega‐3 levels were 0.049, 0.032, and 0.020 g/100 g milk; total conjugated linoleic acid levels were 0.043, 0.023, and 0.019 g/100 g milk; and eicosapentaenoic acid levels were 0.0036, 0.0033, and 0.0025 g/100 g milk. Because of often high per‐capita dairy consumption relative to most other sources of omega‐3 fatty acids and conjugated linoleic acid, these differences in grassmilk can help restore a historical balance of fatty acids and potentially reduce the risk of cardiovascular and other metabolic diseases. Although oily fish have superior concentrations of long‐chain omega‐3 fatty acids, most fish have low levels of α‐linolenic acid (the major omega‐3), and an omega‐6/omega‐3 ratio near 7. Moreover, fish is not consumed regularly, or at all, by ~70% of the U. S. population.

## 1 INTRODUCTION

Nearly half of Americans suffer from one or more diet‐driven chronic conditions including cardiovascular disease (CVD), overweight and obesity, and diabetes (DHHS, 2015; Massiera et al., 2010; FDA, 2015; ARS, 2010). Seven of the 10 leading causes of death in the United States were diet‐related in 2013 (IHME, 2016aa), and none are curable via medical intervention alone, despite health care spending in the United States that is the highest per capita in the world (IHME, 2016ab). These sober facts are among the reasons why there is growing interest in the United States among scientists and consumers in altering diets to prevent or slow the progression of metabolic, cardiovascular, and other chronic diseases.

Potential diet alterations include reducing intakes of omega‐6 (ω‐6) fatty acids (FAs) and increasing intakes of omega‐3 (ω‐3) FAs, thus decreasing dietary ω‐6/ω‐3 ratios. These ratios have become historically high in Western diets during the last century, reaching about 15, compared to estimated evolutionary ratios near 1 (Hibbeln, Nieminen, Blasbalg, Riggs, & Lands, 2006; Simopoulos, 2006). These large changes are due to both increased intakes of ω‐6 FAs and decreased intakes of ω‐3 FAs. Modern grain feeding of farm animals has contributed to these ω‐6 increases and ω‐3 decreases in Western diets.

The more natural FA profile of organic and grass‐fed meat and milk has received much attention in recent years (Średnicka‐Tober et al., 2016a,b). The FA profile in modern meat and milk can be substantially changed by shifting animals from grain‐ or concentrate‐rich rations to diets largely based on grass and legume forages (Butler et al., 2011; Daley, Abbott, Doyle, Nader, & Larson, 2010; O' Callaghan et al., 2016; Schwendel et al., 2015; Stergiadis et al., 2012). This shift increases ω‐3 FAs and conjugated linoleic acid (CLA) and decreases ω‐6 FAs in meat and milk, changes that may help prevent CVD and other chronic conditions (Leikin‐Frenkel, 2016; Hibbeln et al., 2006; Simopoulos, 2006). The magnitude of these changes is markedly greater than most of the nutritional differences between organically and conventionally grown plant‐based foods (Benbrook, Butler, Latif, Leifert, & Davis, 2013; Baranski et al., 2014; Średnicka‐Tober et al., 2016a,b).

There is rising demand for beef and dairy products from grass‐fed cattle. In 2016, natural‐food retail leader Whole Foods Market identified grass‐fed meat and dairy as a top trend, based on consumer interest and rapid sales growth (PR Newswire, 2016; Whole Foods Market Blog, 2015). Three‐quarters of self‐identified natural‐food and organic consumers purchase grass‐fed beef and dairy (Market Lohas, 2016). Similar interests in human health and animal welfare led in 2015 to the marketing in Italy, and later Mexico, of “Latte Nobile” (Noble Milk), produced by cows fed primarily grass and hay (Renna et al., 2015; Lombardi et al., 2014; Associazione Latte Nobile Italiano (http://www.lattenobile.it/).

### 1.1 Launch of grassmilk brand

The Wisconsin‐based cooperative CROPP is the leading U.S. supplier of organic milk. In 2011, CROPP launched a new, whole milk, organic product called Grassmilk.^TM^ This milk comes from cows fed a nearly 100% forage‐based diet. The only exception is certain mineral and energy supplements, such as molasses. In this paper, the term “grassmilk” refers to CROPP's product, and “grass milk” refers to other brands of milk from cows fed a nearly 100% forage‐based ration.

Besides prohibiting grain in cow rations, CROPP sets grassmilk standards for pasture access, supplemental feeds, and animal care (see Appendix for details). Farmers in the grassmilk program receive a price premium of ~15% compared to the organic milk price. CROPP closely monitors the FA content of raw grassmilk, to assure compliance with its minimum requirements of (1) 39 to 41 mg total ω‐3 FA/100 g of milk, depending on geographical region, (2) 26.6 to 32.8 mg total CLA/100 g of milk, and (3) an ω‐6/ω‐3 ratio ≤ 1.2.

The number of farms shipping grassmilk to CROPP processors has grown from five California producers in 2011 to 140 farms throughout the United States at the end of 2016. These 140 farms represented about 9% of CROPP's 1,618 dairy‐farm members (CROPP, 2016). Milk, yogurt, and cheese made from grassmilk are marketed under CROPP's Organic Valley brand. In addition to meeting USDA's organic grazing standard (Rinehart & Baier, 2011), CROPP's grassmilk suppliers may not feed grain or silage from grain crops harvested from fields that have reached the “boot” stage of development (when seed heads form and start to fill out). Nongrain supplements including molasses, alfalfa pellets, sugar beets (chipped or whole), mineral supplements, and kelp are allowed to meet the energy needs of lactating cows and support animal health.

Some supplemental feed is often needed to sustain cow health during months of peak production, or when high‐quality, forage feeds are not available in sufficient quantity. Despite reliance on some supplemental feeds, forage‐based feeds make up the vast majority of annual Dry Matter Intake (DMI) on grassmilk farms.

Farmers in the grassmilk program are also required to document that lactating cows consume over 60% of DMI from pasture during the grazing season (compared to 30% under the USDA organic standard), with a grazing season of at least 150 days (compared to 120 days under federal organic rules). The length of the grazing season can be reduced in cases of extreme drought or other weather events or natural disasters, or by the tolerance of soils to animal traffic.

The nongrazing portion of rations on grassmilk farms must come from conserved, organic, forage‐based feeds, including dried or fermented forages (alfalfa, clover, grass hay, etc.). Cereal crops harvested prior to their boot stage, such as barley, oats, and BMR corn (“brown mid‐rib” phenotypes developed for early silage harvest), can also be fed, as the FA profile of such immature grain crops is similar to widely grown grass species in cow pastures (see “Results and Discussion” for more detail). Harvested feedstuffs are typically preserved by fermentation on‐farm to produce baleage or silage, or stored as dry hay.

The mentioned Noble Milk protocol requires at least 150 days per year of grazing and 70% DMI from pasture and hay throughout the year, with up to 30% DMI from grains and concentrates allowed. Silages, supplements, and genetically modified feeds are prohibited. The milk fat must contain at least 0.25% of total CLA and 0.5% of total ω‐3 FAs, and the LA/ALA ratio must be lower than 4 year round (Renna et al., 2015). Pastures must be diverse, with at least four major plant species, and the quality of hay is monitored with a sensory analysis (Rubino, 2014).

Benbrook et al. (2013) reported substantial improvements in the FA composition of Organic Valley whole, organic milk, compared to whole milk from conventionally managed cows. Based on 12‐month averages, they found higher levels of ω‐3 FAs α‐linolenic acid (ALA) and eicosapentaenoic acid (EPA) of, respectively, +60% and +33%. There was also 18% more total CLA, and less of the ω‐6 FAs linoleic acid (LA) and arachidonic acid (AA) (25% and 17%, respectively), resulting in a 60% lower ω‐6/ω‐3 ratio (5.77 down to 2.28).

As estimated below, most organic milk analyzed in Benbrook et al. (2013) came from cows receiving ~20% of their yearly DMI from grain‐based feeds. On an annual basis, the USDA organic standard technically allows up to ~90% DMI from sources other than grazing (as only 30% of DMI must come from grazing during a minimum of 120 days per year), although on most organic dairies in the United States, forage‐based feeds play a much greater role than is minimally required (CROPP, 2016; Rinehart & Baier, 2011).

Here, we report the added impacts on nutritionally important FA levels when lactating cows are fed a nearly 100% forage‐based diet year round, and we model the impact of these changes on typical U.S. diets. We also compare the impacts of grassmilk dairy products and fish on FA intakes.

## 2 METHODS

Milk FA analyses reported in this study come from CROPP's quality‐control testing of its grassmilk. Bulk‐tank, raw milk samples from each participating farm (140 in 2016) were collected at least bimonthly in sterile plastic bottles, packed with ice‐packs, and shipped overnight to Silliker, Inc., an ISO/IEC 17025 accredited laboratory in Crete, Illinois. It used AOAC method 996.06, as revised in 2001 (AOAC International, 2012), with modified internal standard (C13:0) and temperature program [initial *T* = 100° (no hold), ramp 2°/min to 214° (hold 10 min), ramp 3°/min to 240° (hold 16 min)]. The laboratory used capillary column Supelco SP‐2560, 100 m × 0.25 mm, 0.2 μm film. In units of g/100 g of milk, the laboratory did not quantify individual FA amounts <0.001, but it did quantify those small amounts (if detected) in units of % of total FA, to give the best measure of total FA. This laboratory and its methods and reports are identical to those used in Benbrook et al. (2013). However, in this paper, we report amounts of additional, minor FA that were not reported in the 2013 paper, due to the relatively small number of samples in 2013.

The detected and summed isomers of reported total CLA include *cis*‐9, *trans*‐11 (commonly 75–90% of the total); *trans*‐9, *trans*‐11; *cis*‐9, *cis*‐11; *trans*‐10, *cis*‐12; and *cis*‐11, *trans*‐13 18:2. The reported *trans*‐18:1 includes mainly *trans*‐11 18:1, vaccenic acid.

This study includes FA analyses of 1,163 raw milk samples collected monthly or bimonthly during three full years, 2014–2016. They come primarily from three regions of the United States—Midwest, Northeast, and California. A small group of samples came from the Middle‐Eastern United States beginning in June 2016. For comparison with raw grassmilk, we also report FA results from 69 samples of processed, whole grassmilk, taken from pasteurized, retail containers (not homogenized). These samples were taken in a systematic manner similar in location and season to the raw milk samples. In 2014, there were 22 samples—12 from the Midwest and 10 from California; in 2015, there were 23 samples—five from the Midwest, six from California, and 12 from the Northeast; and in 2016, there were 24 samples—six from the Midwest, six from California, and 12 from the Northeast.

As in the 2013 study (Benbrook et al., 2013), we report averages of three ω‐6/ω‐3 ratios: LA/ALA, ω‐6/ω‐3, and ω‐3/ω‐6, where ω‐6 includes seven FAs (18:2 LA + 18:3 γ‐linolenic (GLA) + 20:2 eicosadienoic + 20:3 8,11,14‐eicosatrienoic + 20:4 arachidonic (AA) + 22:2 docosadienoic + 22:4 docosatetraenoic), and ω‐3 includes 7 FAs (18:3 ALA + 18:4 stearidonic/moroctic + 20:3 11,14,17‐eicosatrienoic + 20:5 EPA + 22:3 docosatrienoic + 22:5 DPA + 22:6 DHA). We include the average ratio, ω‐3/ω‐6, for comparison with other papers that report this inverted ratio.

### 2.1 Statistical analysis

Digital laboratory results were transferred to an Excel spreadsheet and spot‐verified against printed laboratory reports. (The raw data are available from MAL or DRD.) The reported FA concentrations (g/100 g of milk) in 1,163 raw milk samples were inspected for outliers by normal probability and box plots, and five severe, high outliers were excluded, mostly by consensus among DRD, MB, BH, and CMB (18:3 γ‐linolenic = 0.028, *trans*‐18:3 = 0.075, 20:1 = 0.070, 11,14,17–20:3 = 0.040, and 22:6 DHA = 0.025, all in g/100 g of milk). We also removed the corresponding values expressed as a percent of total FAs. An additional four outliers were found and removed only in the values expressed as a percent of total FA: sum of FA = 102.998% (high), sum of saturated FA = 87.690% (high), 18:1 = 0.330% (low), and sum of *cis*‐monounsaturated = 4.89% (low). The removed values represent in each case only 1 in 1,163 samples (<0.1%). No outliers were found in the 69 samples of retail grassmilk.

Means, counts, standard deviations (*SD*s), coefficients of variation (CVs), and standard errors (*SE*s) were calculated in Microsoft Excel. We report *SD*s, CVs, and *SE*s with 1 or 2 significant digits. Because the statistical uncertainty of a mean is measured by its *SE*, we report means to the same number of decimal places as the *SE*s. With sample counts as high as 1,163, SEs and the statistical uncertainty of means can be much smaller than the laboratory precision for individual measurements.

Analyses of annual, monthly, and regional variation in FA concentrations used PROC MIXED of SAS (SAS Institute, 2014). The fixed effects were year of study, region of the United States (California, Midwest, Mideast, and Northeast), and month of sampling, with farm as a random effect for repeated measures. The compound symmetry covariance structure was used, because it resulted in the lowest Akaike information criterion for repeated measures (Littell, Henry, & Ammerman, 1998). We relied on the Satterthwaite correction to adjust the degrees of freedom for unequal variances. All treatment results are reported as least squares means separated by the Tukey procedure with significance declared at *p *<* *.05.

### 2.2 Diet scenarios and LA/ALA ratios

We modeled hypothetical diet scenarios based on those in Benbrook et al. (2013) to test the potential effects of switching whole‐fat dairy products made from conventional milk, to organic milk, and, finally in this study, to grassmilk. For these diets, we calculated overall dietary intakes of LA and ALA, and the LA/ALA ratio, the major determinant of the ω‐6/ω‐3 ratio. We could not calculate the ω‐6/ω‐3 ratio itself, because there is insufficient data on total ω‐6 and ω‐3 FAs in most of the foods in our scenarios. However, the ω‐6/ω‐3 ratio closely tracks the LA/ALA ratio, and both ratios are historically high in most Western diets, due to increased ω‐6 intakes and decreased ω‐3 intakes (Hibbeln et al., 2006; Simopoulos, 2006).

Here, we use the same model diets and assumptions as in the previous report (Table 1 in Benbrook et al., 2013), and add new diet scenarios using dairy products with the FA profile of grassmilk. As in Benbrook et al. (2013), we use full‐fat dairy products (except for yogurt), to quantify the maximum, realistically attainable shift in FA intakes from a switch to grass‐milk‐based dairy products. For yogurt, we use the highest‐fat form generally consumed in the United States, sweetened “low‐fat” yogurt with fruit, containing 1.41 g fat per 100 g. Sweetened whole‐fat yogurt is not usually available.

We modeled diets for a moderately active woman, age 19–30, consuming 2,100 kcal/day. In three main scenarios, 20%, 33%, or 45% of that energy came from fat. Within those scenarios, we constructed diets that contain either moderate amounts of dairy products (three daily servings, as recommended in the *Dietary Guidelines for Americans* (DHHS, 2015), or 50% higher amounts (4.5 servings/day). Whole milk, Cheddar cheese, low‐fat yogurt, and ice cream as a “dairy dessert” were the dairy products included (Table 1 in Benbrook et al., 2013).

For the LA and ALA contents of dairy fat, the previous study used its measured 12‐month average concentrations in conventional and organic milks (Benbrook et al., 2013). For the LA and ALA in nondairy foods, the authors used USDA's standard reference data for 8 common foods to represent “typical‐LA nondairy sources” (USDA, 2015). Those foods averaged 23.23 g LA and 1.841 g ALA per 100 kcal of fat, for an LA/ALA ratio of 12.6. To illustrate the effects of reducing LA intake, they substituted three of the eight foods with similar, low‐LA foods and ingredients (e.g., canola oil instead of soy oil, the major oil used in many foods). These revised eight “low‐LA nondairy sources” averaged 13.84 g LA and 2.731 g ALA per 100 kcal of fat, with an LA/ALA ratio of 5.07.

With these assumptions, the 2013 authors calculated the LA/ALA ratios for 12 diets with typical‐LA nondairy sources (3 fat levels × 2 levels of dairy consumption × 2 types of dairy fat) and for an additional 12 diets with low‐LA nondairy sources. Here, we add to these calculations a 3^rd^ type of dairy fat with the average FA profile found here in 1,163 grassmilk samples.

## 3 RESULTS AND DISCUSSION

We set out to answer two key questions. First, to what extent does shifting lactating dairy cattle to nearly 100% forage‐based feeds alter the FA profile of their milk compared to currently available conventional and organic milks in the United States? The following subsection presents results from 3 years of nationwide sampling, including seasonal and regional variations.

Our second core question is how much can improvements in the FA profile of grassmilk help reverse historically high dietary ω‐6/ω‐3 ratios? We address this question with nutrition modeling results in the third subsection below.

### 3.1 Altering the fatty acid profile of milk

Table 1 shows concentrations of 37 main FAs (quantified amounts >0.001 g/100 g milk) in raw, whole grassmilk, averaged over 3 years (2014–2016), reported as g/100 g of milk and as a percentage of total FAs. For each FA, there are 1,163 values, less the nonquantified samples and any outliers removed (as explained in Methods). See Table 1 footnotes ^a^ and ^b^ for details. The coefficients of variation (CV = *SD*/mean) are a measure of variability among samples.

**Table 1:** Fatty acids in raw, whole grassmilk, 36‐month average, 2014–2016 (1,163 samples)

	g/100 g milk^a^					Percent of total fatty acids^b^				
	Mean	*n*	*SD*	CV (%)	*SE*	Mean	*n*	*SD*	CV (%)	*SE*
Fat measured at farm	4.237	1162	0.49	12	0.014					
Total triglyceride (calculated)	3.881	1161	0.53	14	0.016					
Total fatty acids	3.585	1161	0.48	13	0.014	99.996	1160	0.15	0.1	0.004
Saturated fatty acids										
4:0 butyric	0.09202	1163	0.015	17	0.00045	2.572	1163	0.31	12	0.009
6:0 caproic	0.06967	1158	0.013	18	0.00037	1.940	1158	0.21	11	0.006
8:0 caprylic	0.04088	1160	0.009	21	0.00025	1.136	1160	0.16	14	0.005
10:0 capric	0.0963	1163	0.022	23	0.0007	2.671	1163	0.42	16	0.012
12:0 lauric	0.1106	1162	0.027	24	0.0008	3.066	1162	0.50	16	0.015
14:0 myristic	0.3997	1160	0.07	18	0.0021	11.114	1160	1.1	10	0.032
15:0 pentadecanoic	0.05619	1163	0.012	21	0.00034	1.567	1163	0.24	16	0.007
16:0 palmitic	1.116	1162	0.24	21	0.007	31.00	1162	4.3	14	0.13
17:0 margaric	0.0315	1163	0.007	21	0.00019	0.878	1163	0.13	15	0.004
18:0 stearic	0.3738	1163	0.08	22	0.0024	10.455	1163	2.0	19	0.057
20:0 arachidic	0.006756	1149	0.0016	24	0.000047	0.1878	1149	0.035	19	0.0010
22:0 behenic	0.004552	1155	0.0016	34	0.000046	0.1269	1155	0.040	32	0.0012
24:0 lignoceric	0.002443	1153	0.0007	28	0.000020	0.0682	1153	0.015	22	0.0004
Total saturated^c^	2.399	1163	0.39	16	0.011	66.71	1162	4.2	6	0.12
Monounsaturated fatty acids										
14:1 myristoleic	0.03404	1160	0.0095	28	0.00028	0.944	1160	0.20	22	0.006
16:1 palmitoleic	0.05643	1159	0.014	26	0.00043	1.571	1159	0.33	21	0.010
17:1 margaroleic	0.01052	1157	0.0029	28	0.00009	0.295	1157	0.08	26	0.002
18:1 including oleic	0.7258	1163	0.12	16	0.0034	20.36	1162	2.8	14	0.08
20:1 including gadoleic	0.00724	1161	0.0020	28	0.00006	0.2024	1161	0.05	25	0.0015
Total *cis*‐monounsaturated^c^	0.8352	1163	0.12	15	0.0036	23.41	1162	2.6	11	0.08
ω‐3 fatty acids										
18:3 α‐linolenic, ALA	0.04409	1163	0.011	25	0.00032	1.229	1163	0.26	21	0.008
18:4 stearidonic/moroctic	0.002636	841	0.0010	37	0.000034	0.0729	844	0.025	35	0.0009
20:3 11,14,17‐eicosatrienoic	0.001139	736	0.00036	32	0.000013	0.0306	747	0.009	31	0.0003
20:5 eicosapentaenoic, EPA	0.004132	1157	0.0010	23	0.000029	0.1148	1157	0.021	18	0.0006
22:3 docosatrienoic	0.00114	14	0.00036	32	0.00010	0.028	16	0.015	53	0.004
22:5 docosapentaenoic, DPA	0.005432	1158	0.0012	23	0.000036	0.1519	1158	0.030	20	0.0009
22:6 docosahexaenoic, DHA	0.001064	249	0.0005	50	0.000034	0.0266	258	0.018	67	0.0011
Total ω‐3^d^	0.05645	1161	0.013	23	0.00038	1.573	1161	0.30	19	0.009
ω‐6 fatty acids										
18:2 linoleic, LA	0.04469	1156	0.010	22	0.00029	1.254	1156	0.27	22	0.008
18:3 γ‐linolenic, GLA	0.001100	649	0.00031	28	0.000012	0.0299	670	0.009	31	0.0004
20:2 eicosadienoic	0.001031	717	0.00017	17	0.000006	0.0258	743	0.007	28	0.0003
20:3 8,11,14‐eicosatrienoic (γ)	0.001964	1113	0.0006	32	0.000019	0.0548	1114	0.014	26	0.0004
20:4 arachidonic, AA	0.003453	1131	0.0015	43	0.000045	0.0920	1132	0.043	46	0.0013
22:2 docosadienoic	0.001803	969	0.0007	39	0.000023	0.0502	975	0.017	33	0.0005
22:4 docosatetraenoic	0.00118	91	0.0005	47	0.00006	0.024	108	0.016	70	0.002
Total ω‐6^d^	0.05250	1163	0.012	22	0.00034	1.467	1163	0.31	21	0.009
Total *cis*‐polyunsaturated^c^	0.10885	1161	0.022	21	0.00066	3.045	1161	0.5	17	0.015
*trans* fatty acids										
*trans*‐14:1	0.01335	1162	0.0029	22	0.00008	0.372	1162	0.07	17	0.002
*trans*‐16:1, *trans*‐palmitoleic	0.01964	1159	0.0042	21	0.00012	0.551	1159	0.11	20	0.003
*trans*‐18:1 including elaidic	0.1381	1160	0.05	39	0.0016	3.885	1160	1.5	39	0.045
*trans*‐18:2 octadecadienoic	0.02203	1159	0.008	35	0.00022	0.618	1159	0.21	34	0.006
Total *trans* fatty acids^c^	0.1934	1163	0.06	31	0.0018	5.430	1163	1.7	31	0.049
Conjugated linoleic acid, CLA										
18:2 conjugated, total	0.0498	1163	0.019	38	0.0006	1.403	1163	0.5	38	0.016
Sum										
ALA + CLA	0.0939	1163	0.022	24	0.0006	2.633	1163	0.6	22	0.017
Ratios										
LA/ALA	1.042	1156	0.21	21	0.006	1.042	1156	0.21	21	0.006
ω‐6/ω‐3	0.954	1154	0.18	19	0.005	0.947	1161	0.19	20	0.006
ω‐3/ω‐6	1.083	1154	0.20	18	0.006	1.131	1161	0.6	54	0.018

a For FAs reported in units of g/100 g milk, means and the other statistics are based on quantified amounts ≥ 0.001 g/100 g (samples < 0.001 g/100 g not included). Hence, for minor FAs with *n* substantially <1,163, means are elevated, and other statistics are based on the distribution of samples ≥0.001 g/100 g.

b For units of % of total FAs, means and other statistics have the same properties as noted above for units of g/100 g milk. For a few minor FAs, the laboratory quantified up to 26 more samples in units of % of total FAs than it did in units of g/100 g, increasing the *n*‐values shown here. In rare cases, the *n*‐values differ also by ± 1 due to differences in the number of outliers removed.

c An average of sums reported by the laboratory for each sample. The laboratory sums include minor FAs reported in Table S1 but not tabulated here, so they usually slightly exceed the sum of means for the individual FAs listed here.

d An average of sums of all 7 FAs for each sample. This average is slightly smaller than the sum of means shown for each FA, because some of the latter means are substantially elevated by exclusion of values < 0.001 mg/100 g milk (footnote^a^).

Table S1 shows the same information for 14 minor FAs in grassmilk. Table 2 shows the same information as Table 1 for 69 retail samples of grassmilk taken during 2014–2016. The FA profiles of these samples of processed, whole‐fat grassmilk were measured to determine whether there were any significant changes in the FA profile of grassmilk as a result of processing and pasteurization. For FA concentrations expressed as a percentage of total FAs, the amounts in Tables 1 and 2 are very similar, as expected: The means in Table 2 average 101 ± *SD* 5% of the means in Table 1 (for 33 FAs with *n* > 50% of the analyzed samples). However, for FA concentrations expressed in g/100 g milk, the means in Table 2 average only 75 ± *SD* 4% of the means in Table 1 (for 38 FAs with *n* > 50% of the analyzed samples). These values are <100%, mainly because fat is removed from raw milk to produce retail whole milk with a standardized 3.25% fat content.

**Table 2:** fatty acids in retail, whole grassmilk, 36‐month average, 2014–2016 (69 samples)

	g/100 g milk^a^					Percent of total fatty acids^b^				
	Mean	*n*	*SD*	CV (%)	*SE*	Mean	*n*	*SD*	CV (%)	*SE*
Fat reported by processor	3.384	69	0.14	4	0.016					
Total triglyceride (calculated)	2.758	69	0.44	16	0.053					
Total fatty acids	2.616	69	0.42	16	0.050	100.000	69	0.006	0.01	0.0008
Saturated fatty acids										
4:0 butyric	0.0696	68	0.012	17	0.0014	2.656	68	0.25	9	0.030
6:0 caproic	0.0516	69	0.010	19	0.0012	1.965	69	0.18	9	0.022
8:0 caprylic	0.0306	69	0.006	21	0.0008	1.162	69	0.14	12	0.017
10:0 capric	0.0728	69	0.014	19	0.0017	2.775	69	0.28	10	0.034
12:0 lauric	0.0830	68	0.016	19	0.0019	3.170	68	0.29	9	0.036
14:0 myristic	0.298	68	0.049	17	0.006	11.33	68	0.7	6	0.09
15:0 pentadecanoic	0.0402	68	0.008	20	0.0010	1.538	68	0.18	12	0.022
16:0 palmitic	0.812	69	0.15	18	0.018	31.09	69	3.2	10	0.39
17:0 margaric	0.0225	69	0.0045	20	0.0005	0.858	69	0.09	10	0.011
18:0 stearic	0.276	69	0.06	22	0.007	10.52	69	1.5	14	0.18
20:0 arachidic	0.00496	67	0.0007	15	0.00009	0.1879	67	0.021	11	0.0025
22:0 behenic	0.00338	66	0.0013	37	0.00016	0.126	66	0.042	34	0.005
24:0 lignoceric	0.00191	65	0.0005	24	0.00006	0.0715	65	0.014	20	0.0017
Total saturated^c^	1.758	69	0.29	17	0.035	67.18	69	2.7	4	0.33
Monounsaturated fatty acids										
14:1 myristoleic	0.0244	68	0.005	21	0.0006	0.935	68	0.13	14	0.016
16:1 palmitoleic	0.0409	69	0.010	24	0.0012	1.560	69	0.26	17	0.031
17:1 margaroleic	0.00765	68	0.0017	23	0.00021	0.289	68	0.045	15	0.005
18:1 including oleic	0.506	69	0.12	25	0.015	19.40	69	3.6	19	0.43
20:1 including gadoleic	0.00530	67	0.0013	25	0.00016	0.201	67	0.046	23	0.006
Total *cis*‐monounsaturated^c^	0.585	69	0.13	23	0.016	22.41	69	3.6	16	0.43
ω‐3 fatty acids										
18:3 α‐linolenic, ALA	0.0312	69	0.005	17	0.0006	1.196	69	0.14	12	0.017
18:4 stearidonic/moroctic	0.00206	35	0.0009	46	0.00016	0.076	35	0.030	39	0.005
20:3 11,14,17‐eicosatrienoic	0.001000	35	0.00000	0	0.000000	0.0297	39	0.009	32	0.0015
20:5 eicosapentaenoic, EPA	0.00316	63	0.0006	19	0.00008	0.1208	63	0.020	16	0.0025
22:3 docosatrienoic	0.00100	2				0.040	2			
22:5 docosapentaenoic, DPA	0.00408	65	0.0009	21	0.00011	0.1551	65	0.024	16	0.0030
22:6 docosahexaenoic, DHA	0.00108	12	0.0003	27	0.00008	0.0325	13	0.015	48	0.0043
Total ω‐3^d^	0.0397	69	0.007	19	0.0009	1.515	69	0.20	13	0.024
ω‐6 fatty acids										
18:2 linoleic, LA	0.0332	69	0.007	20	0.0008	1.272	69	0.18	14	0.022
18:3 γ‐linolenic, GLA	0.001031	32	0.00018	17	0.000031	0.0302	37	0.012	39	0.0020
20:2 eicosadienoic	0.00113	31	0.00034	30	0.00006	0.0332	34	0.018	54	0.0031
20:3 8,11,14‐eicosatrienoic (γ)	0.00159	63	0.0005	33	0.00007	0.0602	63	0.017	29	0.0022
20:4 arachidonic, AA	0.00245	65	0.0009	36	0.00011	0.0910	65	0.034	38	0.0043
22:2 docosadienoic	0.00141	54	0.0005	35	0.00007	0.0551	54	0.015	28	0.0021
22:4 docosatetraenoic	0.00150	2				0.031	4	0.030	95	0.015
Total ω‐6^d^	0.0390	69	0.008	20	0.0009	1.490	69	0.22	14	0.026
Total *cis*‐polyunsaturated^c^	0.0783	69	0.014	18	0.0017	3.012	69	0.37	12	0.044
*trans* fatty acids										
*trans*‐14:1	0.00975	69	0.0018	19	0.00022	0.3709	69	0.035	9	0.0042
*trans*‐16:1, *trans*‐palmitoleic	0.01433	69	0.0033	23	0.00039	0.549	69	0.08	14	0.009
*trans*‐18:1 including elaidic	0.120	69	0.11	95	0.014	4.53	69	4.0	89	0.48
*trans*‐18:2 octadecadienoic	0.0156	68	0.0047	30	0.0006	0.596	68	0.16	26	0.019
Total *trans* fatty acids^c^	0.159	69	0.12	74	0.014	6.05	69	4.1	67	0.49
Conjugated linoleic acid, CLA										
18:2 conjugated, total	0.0353	69	0.010	30	0.0013	1.352	69	0.33	24	0.040
Sum										
ALA + CLA	0.0665	69	0.014	20	0.0016	2.548	69	0.35	14	0.042
Ratios										
LA/ALA	1.069	69	0.15	14	0.018	1.070	69	0.15	14	0.018
ω‐6/ω‐3	0.992	69	0.13	14	0.016	0.991	69	0.13	13	0.016
ω‐3/ω‐6	1.026	69	0.14	14	0.017	1.028	69	0.14	14	0.017

a For FAs reported in units of g/100 g milk, means and the other statistics are based on quantified amounts ≥0.001 g/100 g (samples <0.001 g/100 g not included). Hence, for minor FAs with *n* substantially <69, means are elevated, and other statistics are based on the distribution of samples ≥0.001 g/100 g milk.

b For units of % of total FAs, means and other statistics have the same properties as noted above for units of g/100 g milk. For a few minor FAs, the laboratory quantified up to 5 more samples in units of % of total FAs than it did in units of g/100 g milk, increasing the *n*‐values shown here. In rare cases, the *n*‐values differ also by ± 1 due to differences in the number of outliers removed.

c An average of sums reported by the laboratory for each sample. The laboratory sums include minor FAs reported in Table S1 but not tabulated here, so they usually slightly exceed the sum of means for the individual FAs listed here.

d An average of sums of all 7 FAs for each sample. This average is slightly smaller than the sum of means shown for each FA, because some of the latter means are substantially elevated by exclusion of values <0.001 mg/100 g milk (footnote^a^).

Table 3 compares selected FA levels and ratios in organic grassmilk to those in retail conventional milk and organic milk from Benbrook et al., 2013. We incorporated in Table 3 results from the 1,163 samples of raw grassmilk (Table 1) rather than the results from 69 samples of processed grassmilk (Table 2). We did so because the 1,163 samples of grassmilk provide a more accurate, year‐round FA profile of grassmilk than the 69 retail samples. The retail conventional and organic milk samples average ~3.1% total FAs, whereas the raw, grassmilk samples average ~3.6% FAs. During the processing of raw grassmilk, ~0.5% of fat is removed to meet the standard of identity for fat in whole milk. Accordingly, in Table 3, we adjusted the raw grassmilk FA amounts to equal the average total FA content in the retail conventional and organic milks. The last two pairs of columns show the often‐large percentage differences between conventional and adjusted grassmilk, and between organic and adjusted grassmilk.

**Table 3:** Key fatty acids and ratios in whole milk—conventional, organic, and grassmilk (g/100 g milk)

	Conventional^a^	Organic^a^	Grassmilk^b^	Grassmilk (adjusted total FA)^c^	% difference			
					Conventional to adjusted grassmilk (%)	*p* d	Organic to adjusted grassmilk (%)	*p* d
Total fatty acids	3.098	3.108	3.585	3.103	0.2	ns	−0.2	ns
Saturated fatty acids	2.043	2.116	2.399	2.076	1.6	ns	−1.9	ns
14:0 myristic	0.3274	0.3490	0.3997	0.3460	5.7	.002	−0.9	ns
16:0 palmitic	0.8995	0.9344	1.116	0.966	7.4	.0007	3.4	ns
18:0 stearic	0.3559	0.3434	0.3738	0.3235	−9.1	.00000	−5.8	0.001
Monounsaturated fatty acids	0.7944	0.7410	0.8352	0.7229	−9.0	.00000	−2.4	ns
18:1 incl. oleic	0.7074	0.6505	0.7258	0.6282	−11.2	.00000	−3.4	.010
Total ω‐3 Fatty Acids	0.0198	0.0321	0.0565	0.0489	147	.00000	52	.00000
α‐linolenic acid, ALA	0.0159	0.0255	0.0441	0.0382	140	.00000	50	.00000
20:5 eicosapentaenoic, EPA	0.0025	0.0033	0.00413	0.00357	43	.00000	8.3	.0008
22:5 docosapentaenoic, DPA	0.0037	0.0044	0.00543	0.00470	27	.00000	6.8	.004
22:6 docosahexaenoic, DHA	e	e	0.00106^f^	0.00092^f^				
Total ω‐6 fatty acids	0.0948	0.0711	0.0525	0.0454	−52	.00000	−36	.00000
18:2 linoleic, LA	0.0856	0.0639	0.0447	0.0387	−55	.00000	−39	.00000
Total polyunsaturated fatty acids	0.1147	0.1037	0.1088	0.0942	−18	.00000	−9.2	.00000
Total *trans* fatty acids	0.1281	0.1254	0.1934	0.1674	31	.00000	33	.00000
Total CLA	0.0192	0.0227	0.0498	0.0431	125	.00000	90	.00000
LA/ALA	6.272	2.568	1.042	1.042	−83	.00000	−59	.00000
ω‐6/ω‐3	5.774	2.276	0.954	0.954	−83	.00000	−58	.00000
ω‐3/ω‐6	0.219	0.456	1.083	1.083	395	.00000	138	.00000

a From Benbrook et al. (2013).

b From Table 1.

c Adjusted to the mean of conventional and organic.

d Pairwise *p*‐values by 2‐tailed t test; ns, not significant (*p* > .05).

e Not reported because quantifiable amounts ≥0.001 g/100 g milk were found in only 2 of 160 conventional and 4 of 218 organic samples.

f Mean of 249 samples (21% of 1162) with quantified amounts ≥0.001 g/100 g milk. We estimate the average DHA content of all 1162 samples is about 0.0006 g/100 g milk, roughly half the unadjusted amount shown here. For this estimate, we assumed that the 913 unquantified samples contained an average 0.0005 g/100 g milk, half of the minimum quantified amount of 0.001 g/100 g milk.

The *p*‐values in Table 3 are from two‐tailed t tests. These are accurate for individual pairwise comparisons within each of the two pairs of columns considered alone, but they somewhat overstate the statistical significance of differences between pairs of columns, and they do not account for multiple comparisons within columns. However, these caveats are minor in view of the usually extremely small *p*‐values by t test. anova methods are questionable due to the unbalanced data and several years between measurements. Total saturated and monounsaturated FA levels in the adjusted grassmilk show only small percentage differences with those in the conventional and organic milks. But large percentage differences occur in the amounts of total ω‐3 and ω‐6 FAs, and total CLA. The mean level of total ω‐3 in the adjusted grassmilk samples is more than twice that in the conventional samples (up 147%). The shift from organic management to nearly 100% forage‐based diets on grassmilk farms increases the level of total ω‐3 FAs by 52%. In the case of total ω‐6 FAs, the level drops 52% in adjusted grassmilk samples compared to conventional samples and drops 36% from organic to adjusted grassmilk.

The increase in ω‐3 FAs from conventional to adjusted grassmilk, coupled with the decreases in ω‐6 FAs, reduces the ω‐6/ω‐3 ratio from 5.8 in conventional milk to 2.3 in organic milk and 0.95 in adjusted grassmilk. Comparable changes occur in the LA/ALA ratio.

Significantly, lactating cows fed a nearly 100% grass‐ and legume‐based diet produce milk with substantially elevated levels of two long‐chain ω‐3 FAs. Compared to conventional milk, adjusted grassmilk averages 43% more 20:5 EPA and 27% more 22:5 DPA, two of three critical long‐chain ω‐3 FAs. The percent increase in 22:6 DHA cannot be calculated, because there was too little found in the conventional and organic samples tested in 2011–2012. We estimate that the absolute average increase in DHA is about 0.0006 g/100 g of milk (Table 3 footnote ^f^). It is widely agreed that typical Western diets provide insufficient supplies of long‐chain ω‐3 FAs, leading the European Food Standard Agency to recommend at least a doubling of average daily intakes of long‐chain ω‐3 FAs (EPA, DPA, and DHA), especially during pregnancy (EFSA, 2010).

Limited data from four small farms for Noble Milk in Italy show shifts in FA profile qualitatively similar to those in grassmilk, but smaller, as expected given the up to 30% grain and concentrates allowed in cow rations (Lombardi et al., 2014). In Noble Milk, the ratios LA/ALA and ω‐6/ω‐3 are about 30% to 50% higher than in grassmilk. In the summer, Noble Milk reaches the *annual average* level in grassmilk for ω‐3 FAs and almost the annual average for total CLA, but both are substantially lower in other seasons. Total ω‐6 is notably low both in Noble Milk (about as low as in grassmilk) and in Italian conventional milk (about 30% less than U.S. conventional milk reported by Benbrook et al., 2013).

### 3.2 *Trans* fatty acid concentrations

Total *trans* FA concentrations (excluding CLA) were one‐third higher in grassmilk compared to the similar levels in the organic and conventional milks shown in Table 3. Other studies have also found that pasture and forage‐based feeds increase the levels of *trans* FA in milk, mainly *trans*‐18:1, simultaneously with increases in CLA, a group of 18:2 isomers, nearly all containing conjugated *trans* bonds (Daley et al., 2010; Mansson, 2008; Vargas‐Bello‐Pérezz & Garnsworthy, 2013). Increases in total CLA are associated with the high ALA content of forage grasses (Elgersma, 2015), so forage feeding increases not only CLA and ω‐3 FA levels in milk (Slots et al., 2009; Molkentin, 2009, but also *trans* FAs. Increased levels of CLA and ω‐3 FAs in milk have been associated with health benefits (Larsson, Bergkvist, & Wolk, 2005; Smit, Baylin, & Campos, 2010; Stender & Dyerberg, 2003; Vargas‐Bello‐Pérezz & Garnsworthy, 2013).

*Trans* FA has a bad reputation because of evidence that sources from partially hydrogenated vegetable oils strongly increase LDL cholesterol, decrease HDL cholesterol, and have multiple other metabolic effects associated with CVD. These adverse findings are often assumed to apply equally to natural sources of *trans* FAs. However, such assumptions are unwarranted, because there are large differences between the two sources of *trans* FAs. First, industrial sources contain up to 60% *trans* FAs, compared to a maximum of 5 to 8% of FAs in milk (5.4% and 6.0% in Tables 1 and 2) (Stender & Dyerberg, 2003). Second, the distribution of isomers differs greatly in the main *trans* FA in milk, *trans*‐18:1 (about 72% and 75% of total *trans* FAs in Tables 1 and 2). In industrial sources, the position of the *trans* bond has a broad, near‐Gaussian distribution from the 6th to the 16th carbon atom, whereas milk and other ruminant sources peak strongly at the 11th carbon atom, with only small amounts at other positions (Stender & Dyerberg, 2003).

*Trans*‐18:1 with the *trans* bond at the 11th carbon atom is vaccenic acid (VA), the major precursor to CLA (rumenic acid) in milk. At the high range of human intakes, VA has little or no adverse effect on risk factors for CVD (Lacroix et al., 2011). VA in the cow's udder is partially converted to rumenic acid, the major CLA in milk (75% to 90%) (Lock & Bauman, 2004; Tyburczy et al., 2008). Humans are also able to convert some VA in milk to this form of CLA (Lock & Bauman, 2004; Turpeinen et al., 2002; Tyburczy et al., 2008). Despite having a *trans* double bond, rumenic acid has proven benefits in animals, especially anticarcinogenic activity against diverse cancer types (Lock & Bauman, 2004). In humans, there is suggestive support for activity against colon cancer from a large, epidemiological study in Sweden (Larsson et al., 2005) and possibly against breast cancer (Dilzer & Park, 2012).

For these reasons, the FDA exempts CLAs from its definition of *trans* FA for purposes of food labeling (FDA, 2003). Several countries and New York City exempt not only CLAs, but also ruminant *trans* FAs such as VA (Larsson et al., 2005; Table 2).

Motard‐Bélanger et al. (2008) conducted a double‐blind, randomized crossover study of “high” and “moderate” dietary intakes of *trans* FAs from specially produced milk. They concluded that high intakes of these *trans* FAs “may adversely affect cholesterol homeostasis,” but that moderate intakes “that are well above the upper limit of current human consumption have neutral effects on plasma lipids and other cardiovascular risk factors.” Their “moderate” intake was 4.2 g/2,500 kcal, where 4.2 g is the amount of total *trans* FAs in 2.64 kg of retail grassmilk (Table 2), or 10.8 servings of one cup (244 g). The “high” amount was 10.2 g/2,500 kcal, 2.43 times higher than the “moderate” amount and far beyond even exceptionally high levels of dairy product consumption in the United States.

Moreover, there is some evidence of benefits from VA associated with its conversion to CLA (Kuhnt, Degen, & Jahreis, 2016). A recent meta‐analysis included 13 randomized, controlled intervention trials that used dairy products as the primary source of *trans* FA, in amounts as high as 4.2% of energy (10.9 g *trans* FA/2,500 kcal) (Gayet‐Boyer, Tenenhaus‐Aziza, Prunet, & Chardigny, 2014). The authors found that these levels of *trans* FA have no harmful effects on HDL cholesterol, LDL cholesterol, or their ratio.

Hence, there is a clear need to distinguish between natural and industrial sources of *trans* FA, but this will take time and careful reporting, because of long‐standing assumptions to the contrary.

### 3.3 Regional and seasonal differences

Our large, nationwide, 3‐year dataset allows assessment of the regional and seasonal consistency in the impact of nearly 100% forage‐based feed on the FA profile of grassmilk. Table 4 shows modest, but sometimes statistically significant, regional differences in grassmilk composition for total ω‐6 and ω‐3 FA. The highest average levels of ω‐3 FAs in grassmilk came from the Midwest and Northeast (1.60% and 1.58% of total FA), while California had the lowest (1.40%), about a 14% difference. Likewise, the Midwest and Northeast had the two highest average concentrations of total ω‐6 FAs. For total CLA, there are no statistically significant regional differences. Average ratios of LA/ALA and ω‐6/ω‐3 varied by 7% across the 4 regions, but these differences are not statistically significant (*p *>* *.05). There were no regional differences for the major FAs in milk—total saturated and total cis‐monounsaturated FAs (not shown).

**Table 4:** Regional variation in selected grassmilk fatty acids, 2014–2016 (% of total FA)^a^

	California	Mideast	Midwest	Northeast	*SEM*	*p*‐value
Observations	85	54	582	442		
Total ω‐3	1.40^c^	1.434^bc^	1.601^a^	1.575^ab^	0.04	.002
Total ω‐6	1.364^ab^	1.309^b^	1.477^a^	1.495^a^	0.04	.002
Total CLA	1.282	1.165	1.300	1.379	0.07	.09
LA/ALA	1.091	1.022	1.035	1.047	0.03	.62
ω‐6/ω‐3	1.189	1.232	1.206	1.151	0.07	.75

a Least square means. Means within a row without common superscripts are different at *p *<* *.05. Means were evaluated using Tukey's multiple comparisons test.

Some regional and seasonal variation in the FA profile of grassmilk is expected, driven by differences in the quality and in botanical composition of fresh and stored forage (Ravetto Enri et al., 2017). Such variations are often triggered by climatic conditions that are most extreme during extended drought or heavy rains leading to water‐logged soils or flooding. The length of the outdoor grazing period also impacts forage quality, as does management attention to sustaining a proper mix of grass and legume species in pastures (so that high‐quality, immature forages are present in pastures from spring through the fall). The timing of forage harvests and how, and how well, forage‐based feeds are conserved also impact forage composition. Despite all these factors, our results show that CROPP farmers switching to grassmilk standards have consistently improved milk FA composition over the broad range of agronomic and pedo‐climatic conditions found in the United States.

Table 5 reports small but sometimes statistically significant differences between years. The average ω‐6/ω‐3 ratio declined each year from 2014 to 2016, for an overall decline of 6% (*p *<* *.05). This decline results from a 3.2% decline in total ω‐6 FAs and a 1.5% increase in total ω‐3 FAs (*p *>* *.05 for both). Many factors might contribute to these changes over 3 years, including improving management, changing climate or pasture conditions, or the increasing numbers of participating farms.

**Table 5:** Yearly variation in selected grassmilk fatty acids (g/100 g milk)^a^

	2014	2015	2016
Observations	364	370	429
Total ω‐3	0.0519^b^	0.0558^a^	0.0527^b^
Total ω‐6	0.0493^b^	0.0538^a^	0.0477^b^
Total CLA	0.0454	0.0444	0.0453
LA/ALA	1.0583	1.0566	1.0284
ω‐6/ω‐3	0.9888^a^	0.9720^a^	0.9276^b^

a Least square means. Means within a row without common superscripts are different at *p *<* *.05.

Seasonal highs and lows in grassmilk FAs are shown in Table 6, averaged over all 1,163 samples (2014–2016). The ω‐6/ω‐3 ratio peaked in July and bottomed in December, with a variation of 30% from low to high. For ω‐6 and ω‐3 levels, the maximum variation was somewhat less, 21% to 22%. The largest seasonal variation occurred in total CLA concentration, which more than doubled in September compared to April. Saturated and monounsaturated FA levels did not vary significantly by month (not shown).

**Table 6:** Seasonal variations of key fatty acids, 2014–2016 means (g/100 g grassmilk)^a^

	High	Month	Low	Month	High/Low
ω‐3	0.0594	December	0.0486	August	1.22
ω‐6	0.0548	October	0.045	February	1.21
CLA	0.0635	September	0.0310	April	2.05
ω‐6/ω‐3	1.093	July	0.838	December	1.30

a Least square means in region‐and‐year mixed model.

**Figure 1:**
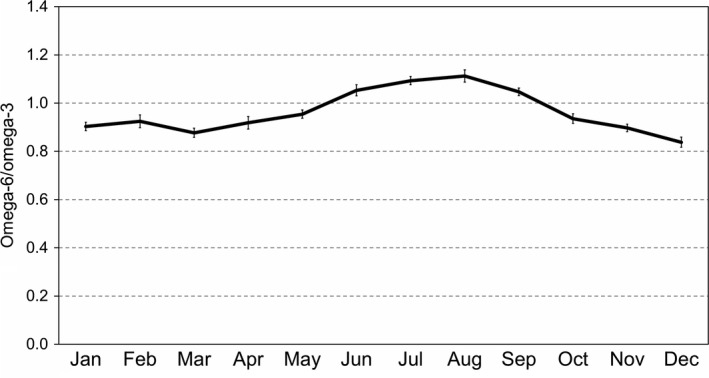
Monthly variation in mean ω‐6/ω‐3 ratio over all geographical regions, 2014–2016 (429 samples). The vertical bars show *SE*s from the least squares analysis

Figure 1 shows the monthly variation in average ω‐6/ω‐3 ratio in all geographical regions during 2014–2016. Figure S1 shows similar plots for the three separate geographical regions with the most samples (sample numbers are 85 for California, 582 for Midwest, and 442 for Northeast). The California region shows notably little monthly variation in ω‐6/ω‐3.

For Noble Milk, reported seasonal variations from four small farms in Italy are considerably larger than in grassmilk for total ω‐3 FAs (twofold larger) and total CLA (nearly threefold larger). However, seasonal variations in LA/ALA and ω‐6/ω‐3 are modest (Lombardi et al., 2014).

### 3.4 Relationship between dairy rations and milk FA profile

All commercial dairy breeds descended from grazing herbivores. The more their diet strays from leafy vegetation, the greater the challenge to maintain gut health. The well‐known effects of grain feeding on milk FAs are caused by alterations in the normal functions of the cow rumen and its microbes (McDonald et al., 2006).

Combining the results of this study of CROPP grassmilk with our prior study of conventional milk and CROPP organic milk (Benbrook et al., 2013), we can estimate quantitative relationships between various levels of grain feeding of dairy cows and the FA composition of their milk. Table 7 shows these relationships for the ω‐6/ω‐3 ratio and the content of total CLA. In addition to Grassmilk, Organic milk, and Conventional milk, we include an estimate for “Minimum Forages” milk from cows with no grazing and maximum amounts of grain. Most dairy cows in the United States now receive a very small share, or none of their annual DMI from grazing (0 to 3%). We estimate that cows under “Minimum Forages” management get approximately 60% of DMI from corn silage and concentrate feeds, and 40% from dry or fermented alfalfa hay in a “total mixed ration” (mostly chopped, dry alfalfa hay). More commonly in the United States, cows under Conventional management receive somewhat less corn plus concentrate feeds (47%) and somewhat more stored forage (50%), with about 3% of annual DMI from grazing (total forages, 53%).

**Table 7:** Estimated average daily dry matter intake from grazing, forage, and grain under four management systems: impacts on ω‐6/ω‐3 and total CLA in retail whole milk

Management system	Average daily dry matter intake (DMI)					Milk fatty acids	
	In season	Annual basis				ω‐6/ω‐3	CLA (g/100 g)
	Grazing (%)	Grazing (%)	Stored forages (%)	Grazing plus forages (%)	Grains and concentrates (%)		
Minimum forages	0	0	40	40	60	8.0	0.010
Conventional	6	3	50	53	47	5.8^a^	0.019^a^
Organic^b^	56	28	52	80	20	2.3^a^	0.023^a^
Grassmilk^b^	80	42	58	100	0	0.95	0.043

a Benbrook et al. (2013).

b Estimated from annual pasture and lactating cattle feed surveys by CROPP cooperative.

According to CROPP records, cows under Organic management on its farms receive about 56% of daily DMI from pasture during an average 183‐day season and hence about 28% of their annual DMI from grazing. On CROPP farms producing Grassmilk, pasture accounts for an average 80% of DMI over a 190‐day grazing season, or 42% of annual DMI. Stored, forage‐based feeds add nearly 52% of daily DMI on Organic farms, and 58% on Grassmilk farms, bringing their totals from forage‐based feeds to, respectively, about 80% and nearly 100% of DMI.

In the milk from these four management systems, the ratios of ω‐6/ω‐3 decline from an estimated 8 with Minimum Forages to measured values of 5.8 in Conventional milk, 2.3 in Organic milk, and 0.95 in Grassmilk (Table 7). Simultaneously, the annual average amounts of total CLA in conventional to retail grassmilk increase about fourfold from about 0.010 to 0.043 g/100 g milk. For total CLA, the impact of pasture and forage feeding appears to increase as their proportion of annual DMI increases beyond 80%, an observation that deserves further exploration.

In grain crops, stage of growth impacts the FA composition of feedstuffs in cow rations, as well as the FA profile of milk (Darby et al., 2013; Darby et al., 2010; Duvick et al., 2006). Supplemental Text S1 and Table S2 compare the FAs in common forage grass and legume crops with those of several cereal crops at various stages of maturity.

### 3.5 Nutrition modeling of grass milk effects on dietary LA/ALA ratios

Tables 1, 2, 3 show that increasing forage‐based feeds in rations for lactating cows can significantly alter the FA profile of milk; however, a key question remains. Will consumption of dairy products from cows fed all, or mostly, forage‐based feeds have a meaningful impact on human intakes of FAs, and potentially on public health?

To address this question, we modeled total LA and ALA intakes in the daily diet of a moderately active 19‐ to 30‐year‐old women across 36 diet scenarios—18 diets with typical, high‐LA foods such as regular margarine and other foods containing soy oil, and 18 mostly identical diets in which three foods lower in LA content were substituted (e.g., pita chips instead of corn chips, and margarine made with canola oil instead of soy oil).

The 18 scenarios in each of these two cases (high‐ and low‐LA intakes) entailed three levels of fat intake (20%, 33%, and 45% of total energy), two levels of dairy product consumption (3 and 4.5 servings/day), and three variations of dairy fat (from cows managed under the conventional, organic, and grassmilk systems discussed here, with their varying reliance on grazing and forage rations shown in Table 7).

Our modeling focuses on total intakes of LA and ALA and the LA/ALA ratio (rather than ω‐6, ω‐3, and ω‐6/ω‐3), because the USDA does not publish sufficient and reliable data on the total ω‐6 and ω‐3 contents of many common foods. But for many foods, it does report reliable (fully differentiated) contents of LA and ALA. LA and ALA are, respectively, by far the major dietary ω‐6 and ω‐3 FAs in nearly all foods, so dietary ratios of LA/ALA are a reliable proxy for dietary ω‐6/ω‐3 ratios.

We assess the degree to which each of the 36 dietary scenarios reduces the LA/ALA from the baseline level of 11.3 for 3 servings/day of conventional milk, typical‐LA sources of nondairy fat, and 33% of energy from fat. The lower the ratio, the greater the body's ability to convert dietary ALA to the essential, longer‐chain ω‐3 FAs. This conversion is most important for pregnant and lactating women and for those who consume little or no oily fish (Brenna, 2002; Burdge & Calder, 2005). Oily fish are, per serving, superior sources for EPA and DHA, but even oily fish do not contain enough ALA to significantly alter dietary ratios of LA/ALA or ω‐6/ω‐3 (Benbrook et al., 2013; USDA, 2015).

Table 8 gives sample results from our nutrition modeling calculations for four diets with typical intakes of total fat (33% of energy) and dairy fat with the FA profile of adjusted grassmilk. These four diets include those with moderate (recommended) (DHHS, 2015) dairy intake (3 servings/day) and high dairy intake (4.5 servings/day), with either typical‐LA or low‐LA sources of nondairy fat. For these four diets, the table shows the dairy and nondairy contributions to dietary intakes of LA and ALA, the LA/ALA ratio and changes in this ratio relative to the baseline ratio of 11.33 for recommended intakes of conventional dairy products (Benbrook et al., 2013). Thus, it shows the impact on dietary LA/ALA ratios of switching from conventional to grassmilk dairy products for these four diets. We performed similar calculations for corresponding diets with low and high amounts of total dietary fat (20% and 45% of energy).

**Table 8:** LA and ALA contributions to average‐fat diets with grassmilk dairy fat and typical‐LA and Low‐LA nondairy fat sources^a^

	LA from dairy fat (g)^b^	ALA from dairy fat (g)^b^	LA from other fat (g)^c^	ALA from other fat (g)^c^	Total LA (g)	Total ALA (g)	Total LA/Total ALA ratio
Typical‐LA nondairy fat sources							
Moderate dairy intake	0.41	0.41	9.91	0.79	10.32	1.19	8.64
High dairy intake	0.62	0.62	5.77	0.46	6.39	1.07	5.95
Low‐LA nondairy fat sources							
Moderate dairy intake	0.41	0.41	5.90	1.17	6.32	1.57	4.01
High dairy intake	0.62	0.62	3.44	0.68	4.06	1.29	3.14

a This table extends Table 3 in Benbrook et al., 2013 to include grassmilk. The modeled dairy servings are in Table 1 of that paper. In it, the baseline LA/ALA ratio is 11.33, for moderate consumption of conventional dairy fat.

b Based on LA, ALA, and total FA from Table 1, 8.79 kcal/g dairy fat, and 0.933 g milk FA/g dairy fat. For example, LA 0.41 = 313 kcal (2013 Table 1)/8.79 × 0.0447/3.585 × 0.933.

c Based on 23.23 g LA and 1.841 g ALA per 100 kcal nondairy fat and 8.90 kcal/g nondairy fat For example, LA 9.91 = 380 kcal (2013 Table 1)/8.90 × 23.23/100. Corresponding calculations for low‐LA nondairy fat use 13.84 g LA and 2.731 g ALA per 100 kcal nondairy fat.

For a diet with typical total dietary fat (33% of energy), moderate dairy servings, and typical‐LA sources of nondairy fat, Table 8 shows that a switch from conventional to adjusted grassmilk dairy products would decrease the overall dietary LA/ALA ratio by 2.68 to 8.64 from the baseline ratio of 11.33. Adding 1.5 servings/day, for a total of 4.5 servings/day of dairy products, would further lower the LA/ALA ratio to 5.95—a total drop of 5.37. These are substantial decreases. For corresponding diets with low‐LA sources of nondairy fat, the reductions in LA/ALA ratio are even larger, by 7.31 and 8.19, respectively, for moderate and high consumption of dairy products.

As we discuss below, reductions in dietary LA/ALA ratios of this magnitude seem of potential public health significance. Much of the reductions can be achieved with grassmilk dairy products alone, without reducing intakes of nondairy LA. The opportunity to reduce total dietary LA/ALA ratios from 11.33 to as low as 3.14, and without major changes in dietary patterns, seems notable to us. In our model diets, there are no changes in most foods, including French fries, chocolate chip cookies, chicken, pork, and beef. The modeled food choices represent an attainable option to improve FA intakes in ways that will likely reduce the risk for cardiovascular and other metabolic disorders, at least for some individuals. Many other factors—genetics, age, health status, and environmental exposures—will interact in determining the magnitude of such impacts (Simopoulos, 2006).

**Figure 2:**
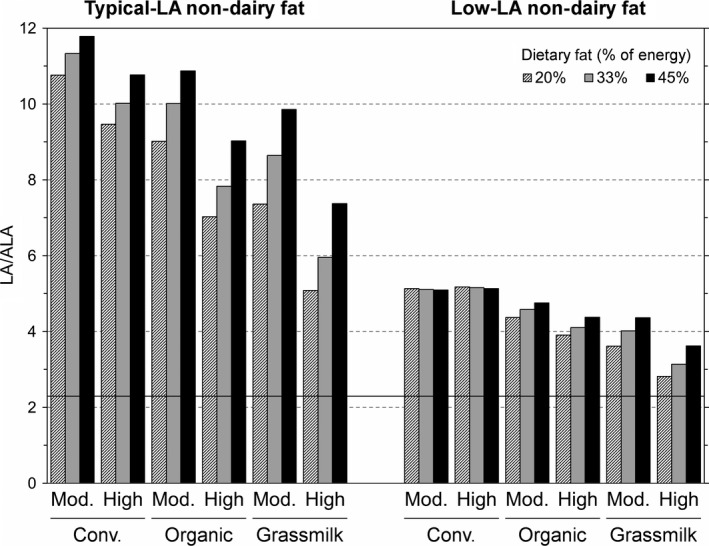
Decreases in dietary LA/ALA ratios for an adult woman consuming two levels of conventional, organic, and grassmilk dairy products and two types of nondairy fat. The diets contain moderate “Mod.”(3 servings/day) or “High” (4.5 servings/day) amounts of dairy products made from conventional (“Conv.”), “Organic,” or “Grassmilk,” in the contexts of total fat contributing 20%, 33%, or 45% of energy, and nondairy fat containing typical amounts of LA (left side) or low amounts of LA (right side)

Figure 2 shows the full results of our nutrition modeling, including diets with low and high intakes of total dietary fat (20% and 45% of energy). For diets with typical‐LA nondairy fat sources (left side of Figure 2), the decreases in dietary LA/ALA ratios are enhanced in the diets with only 20% of energy from fat and attenuated in high‐fat diets. For diets with low‐LA nondairy fat sources (right side of Figure 2), there is little dependence on the overall level of dietary fat, but the reductions in dietary LA/ALA ratio are much larger, including even with conventional dairy fat. Organic and grassmilk dairy fat have the most impact on diets with typical‐LA nondairy fat, compared to diets with low‐LA nondairy fat.

For typical diets with 33% of energy from fat and typical‐LA nondairy fat (left side of Figure 2), switching from moderate amounts of conventional to high amounts of grassmilk dairy products reduces the LA/ALA ratio from 11.33 to 5.95, a 47% reduction. The same switch for diets with low‐LA nondairy fat decreases the LA/ALA ratio from 5.11 to 3.14.

LA and ALA are essential human nutrients, but they both complement and compete with each other, and their dietary ratio matters. They are elongated by parallel and competing pathways to, respectively, AA (from LA) and EPA (from ALA), which in turn are converted into eicosanoids that regulate many body functions. Eicosanoids derived from AA are proinflammatory and thrombogenic, and several have been linked to carcinogenesis, whereas those derived from ALA tend to suppress inflammation, thrombosis, and carcinogenesis, especially when the ω‐6/ω‐3 ratio approaches 1 (Larsson, Kumlin, Ingelman‐Sundberg, & Wolk, 2004).

Thus, a large excess of dietary LA compared to ALA can increase the risk of CVD, cancer, and other diseases (Burdge & Calder, 2005; Ramsden, Hibbeln, Majchrzak, & Davis, 2010; Siri‐Tarino, Chiu, Bergeron, & Krauss, 2015). For some, and perhaps most people in the United States, high‐LA intakes reduce the quantity of ALA converted to EPA and its related eicosanoids and also reduce the conversion of ALA to DHA.

DHA is independently important, because it is required in the development of the infant brain and ocular system (Ailhaud, Massiera, Alessandri, & Guesnet, 2007; Donahue et al., 2011), as discussed further below.

Impaired conversion of ALA to EPA and DHA is of considerable concern in the United States, because most Americans do not consume adequate fish to meet the recommended average intake of 250 mg/day of EPA + DHA (DHHS, 2015; EPA, 2002). Hence, they must partly rely on dietary intake of EPA and DHA from meat and dairy products or supplements. Indeed, in the late 1990s, over 70% of Americans age 18 or older consumed no fish and shellfish (EPA, 2002).

### 3.6 Contribution of grass milk dairy products and fish to fatty acid intakes

Oily fish are the ultimate, direct source of the long‐chain ω‐3 PUFAs, EPA, DPA, and DHA. DHA is present at very low concentrations in other foods, including grass milk, but it plays a vital role in the development of an infant's and child's brain, eyes, and nervous system (Bondi et al., 2013; Moon et al., 2013; Ryan et al., 2010). For the 70% of Americans who consume essentially no fish, the efficiency of conversion of ALA to long‐chain ω‐3 FAs is critically important, especially for those with elevated need, such as growing children and women who are pregnant or breastfeeding. For this conversion, ALA from dairy products and other foods plays dual roles. First as a precursor to EPA, DPA, and DHA, and second by decreasing the LA/ALA ratio, and hence the tendency of LA to capture and utilize the enzymes needed to convert ALA to long‐chain ω‐3 FAs.

Although high in long‐chain ω‐3 FAs, oily fish do not contain significant amounts of either LA or ALA, and for this reason, fish consumption does not significantly impact overall dietary ratios of LA/ALA or ω‐6/ω‐3. Benbrook et al. (2013) used USDA data on the FA contents of seven commonly consumed fish species (canned tuna, tilapia, halibut, sockeye salmon, catfish, trout, and Atlantic salmon) to calculate the amounts of LA, ALA, EPA, DPA, and DHA from 8 ounces of fish per week, the amount recommended in the *Dietary Guidelines for Americans* (DHHS, 2015). This weekly amount of the 7 fish species supplies between 1 (canned light tuna) to 58 mg/day (Atlantic salmon) of ALA, with an average of 20 mg/day (Table 4 in Benbrook et al., 2013). This daily amount of ALA is small compared to the 137 mg in 1.5 cups of grassmilk, or the 162 mg in a 1.5‐ounce serving of cheddar cheese made from grassmilk (see below).

Table 9 shows the amounts of key FA from grassmilk dairy products in our dietary modeling. These amounts complement the data presented in Table 3 of Benbrook et al. (2013) for conventional and organic dairy products. Table 9 also shows the FA content of the 7 commonly consumed fish mentioned above. In addition to these recommended amounts of dairy and fish (DHHS, 2015), Table 9 also shows the FA content of the lower, actual per‐capita consumptions of dairy products (270 g/day) and fish (9.1 g/day). Actual, average per‐capita intakes are 28% of recommended for fish and 42% of recommended for dairy products (Lin, Variyam, Allshouse, & Cromartie, 2003).

**Table 9:** Daily fatty acid content of recommended and per‐capita intakes of dairy products and fish

Dairy or fish sources of fatty acids						Daily fatty acid content (mg)							
	Portion size^a^	Dairy servings/portion	Daily amounts that supply recommended 3 dairy servings			ALA	LA	LA/ALA ratio	Long‐chain ω‐3 fatty acids				
			Portions^a^	Dairy Serv.	Weight (g)				EPA	DPA	DHA^b^	EPA +DPA	EPA + DPA + DHA
Dairy fat from grassmilk^a^													
Whole milk	1 cup	1.0	1.5	1.5	366.0	136.5	138.4	0.954	12.8	16.8	1.9	29.6	31.5
Cheddar cheese	1.5 oz.	1.0	1.0	1.0	42.53	161.7	163.9	0.954	1.5	2.0	0.2	3.4	3.7
Yogurt, low‐fat with fruit	6 oz.	0.5	1.0	0.5	170.1	27.5	27.9	0.954	5.9	7.8	0.9	13.8	14.6
Ice cream	0.5 cup	–	–	–	66.0	84.1	85.2	0.954	2.3	3.0	0.3	5.3	5.7
Totals				3.0	644.6	409.9	415.4	0.954	22.5	29.6	3.3	52.1	55.4
Scaled to U.S. per‐capita daily intake of milk, cheese and other^c^					270	171.7	174.0	0.954	9.4	12.4	1.4	21.8	23.2
	**Daily Portion**		**Daily amount that supplies recommended 8 Oz. Fish/week**										
			**Portions**		**Weight (g)**								
Fat from fish													
Mean of 7 common species^d^	1.143 oz		1.0		32.4	19.6	138	6.5	89.4	37.2	155	127	282
Scaled to U.S. per‐capita daily consumption of finfish^e^			–		9.12	5.5	38.8	6.5	25.2	10.5	43.6	35.6	79.3

a Same foods and portions as in the modeled diets in (Benbrook et al., 2013; Table 1) and Table 8. Calculations use daily serving weight, Table 1 amounts per 100 g, 0.933 g FA per g dairy fat, and the following USDA data: whole milk 244 g/cup and 3.25% fat, cheese 33.14% fat, low‐yogurt 1.41% fat, vanilla ice cream 11.1% fat.

b Using an estimated average DHA content of 0.0006 g/100 g milk, roughly half of the 0.00106 in Table 1 for the 249 highest samples. For this estimate, we assumed that the 913 unquantified samples contained an average 0.0005 g/100 g milk, half of the minimum quantified amount of 0.001 g/100 g milk.

c Table 5 in (Lin et al., 2003).

d Table 4 in (Benbrook et al., 2013) (average of canned tuna, tilapia, halibut, sockeye salmon, catfish, trout, & Atlantic salmon).

e Table 1 in (EPA, 2002).

Based on average per‐capita consumption of dairy products and fish, grassmilk dairy products would supply 31 times more ALA than fish, 4.5 times more LA, 37% as much EPA, 1.2 times more DPA, but only about 3% of the DHA. Grassmilk dairy products supply 29% as much total long‐chain FA (EPA + DPA + DHA) as fish, with a much lower overall LA/ALA ratio (0.95 versus 6.5).

## 4 CONCLUSIONS

We find that nearly 100% grass‐ and legume‐based feeding of lactating dairy cows typically yields milk fat with ratios of LA/ALA and ω‐6/ω‐3 close to 1, compared to 5.8 for milk from cows on conventionally managed farms, and 2.3 for typical (but not nearly 100% grass‐fed) organic dairy farms. Our dietary modeling scenarios show that replacing recommended daily servings of conventional dairy products with grassmilk products and avoiding some foods high in LA could substantially decrease historically high dietary ratios of LA/ALA (and thus ω‐6/ω‐3 ratios) from current values of >10 to as low as 3.1. Such decreases have several potential health benefits, including an enhanced ability to convert dietary ALA to the long‐chain ω‐3 FAs EPA, DPA, and DHA. These nutrients are typically not consumed at recommended levels (DHHS, 2015), and are especially needed during pregnancy and lactation, by children, and by the majority of Americans who eat little or no fish.

Because of the widely varying FA profile of dairy products depending on production systems, coupled with large variations in their fat content (whole, reduced fat, and fat free), the widely disseminated promotional claim “milk is milk” (Dairy Reporter, 2003) is hard to square with the nature of dairy products currently sold and consumed in the United States and elsewhere.

Shifting lactating dairy cows to rations containing substantial portions of forage‐based feeds and less grain dramatically decreases the amounts of LA in milk, while also elevating levels of ALA, long‐chain ω‐3 FAs, and total CLA. These attainable shifts in the FA profile of milk and dairy products are one of several practical ways to potentially improve the quality of American diets. The shifts can be accomplished with existing dairy industry infrastructure and with likely modest impact on food expenditures after a transition period.

Improved messages from government dietary recommendations (Nissen, 2016) and food labeling reforms should, over time, increase consumer demand for grass‐fed beef, milk, and other livestock products. Differentiating more clearly between the FAs implicated or not implicated in the risks for obesity, CVD, and metabolic syndrome will be an additional important step forward.

Further research is needed to determine realistically attainable shifts in FA consumption in the wide diversity of diets across the U.S. population and to assess the cost of alternative paths toward healthier fat intakes. Likewise, further research is needed to identify profitable and scalable changes in livestock feed rations and food manufacturing that will lower dietary ω‐6/ω‐3 ratios and increase intakes of long‐chain ω‐3 FAs and CLA. Improved understanding of the relationship between fat quality and health outcomes will help guide livestock and dairy farmers, the food industry, government agencies, scientists, and physicians searching for promising ways to promote public health.

## ETHICAL STATEMENTS

This study does not involve any human or animal testing. Regarding conflicts of interest, CROPP Cooperative sells grassmilk via its Organic Valley brand. MAL is the Executive Director of Research & Development and Quality Assurance at CROPP Cooperative. LP and SA‐C are on the research and technical services staff of CROPP Cooperative. BJH is faculty supervisor of the University of Minnesota West Central Research and Outreach Center's organic dairy, which markets its milk through CROPP Cooperative and Organic Valley. CMB was Chief Scientist of The Organic Center, 2005–2012, funded in part by CROPP Cooperative; DRD was a consultant to same center, 2011–2012. CMB was program leader for the Measure to Manage program at Center for Sustaining Agriculture and Natural Resources, Washington State University, 2012–2015, for which CROPP was a funder; DRD was a consultant to the same program, 2012–2015.

## APPENDIX 1

### 1.1

**Grass milk standards and oversight**

In 2016, a coalition of dairy industry and certification organizations defined a broad national standard for “100% Grass‐fed dairy” (AGA, 2016). These organizations included Pennsylvania Certified Organic, American Grassfed Association, Northeast Organic Farming Association [New York (NOFA‐NY, 2016) & Vermont chapters], Maple Hill Creamery, and CROPP Cooperative. That coalition adopted CROPP's Grassmilk standards as part of its consensus standard. CROPP's internal standards currently comply with the national consensus, and the cooperative continues to take an active role in solidifying the language and certification requirements associated with nearly 100% grass‐fed dairy claims.

**Feeding requirements**

In addition to the dry hay and fermented hay feeds that are allowed on grass milk farms, CROPP's grassmilk standard allows the feeding of preboot cereal crops. These plants do not contain mature seeds, only plant foliage and stem material. The distinction between grain and foliage from preboot cereal grain crops is based on starch content. Starch is associated with an increase in ω‐6 FAs in the feed and in the resulting milk.

Some farmers transitioning cows to nearly 100%‐forage diets, or managing grass milk herds, need the option to include preboot cereal crops in their conserved feeds. Such feeds increase the energy content of the ration and help sustain cow body condition. They help minimize ω‐6 FAs in the ration and protect the integrity of “no grain” claims in grass milk marketing.

**Animal‐care requirements**

Beyond fat quality, consumers are also increasingly interested in animal welfare. A variety of indicators associated with animal welfare and herd health are finding their way into certification requirements. These metrics include the body condition of lactating cows, outdoor access, use of antibiotics and hormones, physical alterations, lameness, living conditions, and sources of animal stress.

CROPP grassmilk farmers are subject to the same animal‐care reporting and herd‐health verifications that are used throughout the cooperative. These include the National Milk Farmers Assuring Responsible Management (FARM) requirements for body condition, lameness, access to outdoors and water, ventilation, and handling. Some CROPP animal‐welfare requirements go beyond those imposed by FARM, such as prohibition of oxytocin, a drug used to assist in calving.

On‐site audits are performed by staff animal‐care specialists and qualified field staff at least every third year throughout a farm's transition and participation in the program. Audit follow‐up addresses any concerns and sets forth required improvements. Routine visits by CROPP staff also help track and troubleshoot any problems unique to a given farm or region.

Expert advice is available to grassmilk farmers to help them improve their forage quality and production levels, cow health, and profitability. Regular testing of bulk‐tank milk FA levels is used to monitor compliance with the nearly 100% forage‐based feed requirement.

**Challenges and benefits of increasing reliance on forage‐based feeds**

Increasing forage‐based feeds usually reduces milk production. Across farms of all sizes in 2014, rolling‐herd‐average (RHA) 305‐day milk production was 14,513 lb/cow on grazing farms, 14,758 lb on organic farms, and 21,862 lb on conventional farms (APHIS, 2014).

Within CROPP, organic dairies feeding ~20% of DMI from grains have RHAs in the range 14,000 to 18,000 lb/cow, while most grassmilk operations achieve RHAs in the range 6,000 to 16,000 lb/cow. During the spring and early summer, cows in early lactation tend to produce more milk, making it difficult to meet energy needs, especially without a supplemental energy source (e.g., molasses).

Increased reliance on grazing and forage‐based diets requires careful management of soil fertility, pasture composition, forage production, and animal health (especially locomotion). Annual and perennial forage crops are managed collectively throughout the year to provide for both grazing and conserved winter feed. The extreme reliance on pasture and conserved forages may make these farms less resilient in the face of prolonged drought conditions. It remains to be seen whether availability and cost of high‐quality, off‐farm conserved forage put these farms at increased risk during periods of prolonged drought, leading to near‐complete forage crop/pasture losses.

In addition to challenges, grass milk farmers receive benefits. These include a price premium (about 15% on CROPP farms), generally reduced feed costs, and protection from price spikes in organic grains and grain‐based supplements. Agronomically, these farms generally have more tolerance for wet springs, which can delay row‐crop farming practices and increase weed management challenges.

Environmental benefits also accompany the shift to greater reliance on forage‐based feeds and the increased acreage in perennial grass communities. Soil health tends to improve, because of reduced tillage and year‐round perennial grass cover. This improvement in soil health generally results in reduced soil erosion and less sediment and nutrient run‐off into local watersheds compared to conventional tillage, or conservation tillage‐based crop rotations.
