# Supplementary material for: Enhancing the fatty acid profile of milk through forage‐based rations, with nutrition modeling of diet outcomes
Source: Food Sci Nutr. 2018 Feb 28;6(3):681–700. doi: 10.1002/fsn3.610 (PMC5980250; doi:10.1002/fsn3.610)
Supplement: Supplementary file 2 [file FSN3-6-681-s002.docx]

| **Table S1. Minor Fatty Acids in Raw, Whole Grassmilk, 36-Month Average, 2014-2016 (1,163 Samples)** | | | | | | | | | | |
| --- | --- | --- | --- | --- | --- | --- | --- | --- | --- | --- |
|  | **g/100 g milk*** | | | | | **Percent of total fatty acids†** | | | | |
|  | **Mean** | **n** | **SD** | **CV** | **SE** | **Mean** | **n** | **SD** | **CV** | **SE** |
| 5:0 pentanoic (valeric) |  | 0 |  |  |  |  | 0 |  |  |  |
| 7:0 heptanoic (enanthic) |  | 0 |  |  |  |  | 0 |  |  |  |
| 9:0 nonanoic (pelargonic) |  | 0 |  |  |  |  | 0 |  |  |  |
| 11:0 undecylic | 0.0080 | 1 |  |  |  | 0.217 | 1 |  |  |  |
| 12:1 dodecenoic | 0.00224 | 485 | 0.0009 | 39% | 0.00004 | 0.0611 | 486 | 0.020 | 33% | 0.0009 |
| 15:1 pentadecenoic |  | 0 |  |  |  | 0.0130 | 1 |  |  |  |
| *trans*-18:3 octadecatrienoic | 0.001037 | 488 | 0.00019 | 18% | 0.000009 | 0.02548 | 504 | 0.007 | 29% | 0.00032 |
| *trans*-20:1 eicosenoic | 0.001059 | 137 | 0.0006 | 52% | 0.000047 | 0.0209 | 168 | 0.017 | 84% | 0.0013 |
| 20:3 5,8,11-eicosatrienoic | 0.00425 | 4 | 0.0010 | 23% | 0.00048 | 0.114 | 4 | 0.021 | 18% | 0.010 |
| 21:0 heneicosanoic | 0.001604 | 467 | 0.0006 | 36% | 0.000027 | 0.0404 | 517 | 0.016 | 40% | 0.0007 |
| 22:1 docosaenoic (erucic) | 0.001143 | 266 | 0.0006 | 55% | 0.000039 | 0.0257 | 297 | 0.020 | 79% | 0.0012 |
| *trans*-22:1 docosaenoic (brassidic) | 0.00129 | 7 | 0.00049 | 38% | 0.00018 | 0.0295 | 8 | 0.017 | 59% | 0.006 |
| 23:0 tricosanoic | 0.00149 | 75 | 0.0006 | 40% | 0.00007 | 0.0377 | 83 | 0.018 | 47% | 0.0019 |
| 24:1 Tetracosaenoic (nervonic) | 0.001010 | 289 | 0.00010 | 10% | 0.000006 | 0.02029 | 331 | 0.007 | 32% | 0.00036 |
| * For fatty acids reported in units of g/100 g milk, means and the other statistics are based on quantified amounts ≥ 0.001 g/100 g (samples < 0.001 g/100 g not included). Hence, for minor FAs with n substantially less than 1163, means are elevated, and other statistics are based on the distribution of samples ≥ 0.001 g/100 g. | | | | | | | | | | |
| † For units of % of total FAs, means and other statistics have the same properties as noted above for units of g/100 g milk. For these FAs the lab quantified up to 50 more samples in units of % of total FAs than it did in units of g/100 g, increasing the n-values shown here. In rare cases the n-values differ also by ± 1 due to differences in the number of outliers removed. | | | | | | | | | | |
