# Supplementary material for: Enhancing the fatty acid profile of milk through forage‐based rations, with nutrition modeling of diet outcomes
Source: Food Sci Nutr. 2018 Feb 28;6(3):681–700. doi: 10.1002/fsn3.610 (PMC5980250; doi:10.1002/fsn3.610)
Supplement: Supplementary file 3 [file FSN3-6-681-s003.docx]

| **Table S2. Fatty Acid Composition of Common Dairy Cow Forage, Cereal, and Grain Feeds (Percent of Total Fatty Acids)** | | | | | |
| --- | --- | --- | --- | --- | --- |
|  | **Major Saturated FA, Palmitic (16:0)** | **Major Monounsaturated FA, Oleic (18:1)** | **ω-6 or LA** | **ω-3 or ALA** | **LA/ALA or ω-6/ω-3** |
| **Forages** |  |  |  |  |  |
| Alfalfa* | 24.7 | 5.1 | 21.8 | 40.2 | 0.54 |
| Timothy (early flowering)* | 18.9 | 6.5 | 22.1 | 47 | 0.47 |
| Annual ryegrass* | 17.5 | 5.1 | 15.1 | 57.4 | 0.26 |
| Red clover* | 18.8 | 8 | 22.9 | 43 | 0.53 |
| **Cereals** |  |  |  |  |  |
| Corn silage† | 18 | 19 | 48 | 8 | 6 |
| Corn (grain)‡ | 9.8 | 22.7 | 64.3 | 1.3 | 49 |
| Oats (vegetative)§ | 21.5 | 13.0 | 26.3 | 30.8 | 0.85 |
| Oats (grain)* | 16.6 | 33.9 | 38.3 | 1.4 | 27.4 |
| Barley (vegetative)║ | 21 | 11.4 | 32.1 | 28.6 | 1.12 |
| Barley (grain)* | 22.3 | 13.4 | 53.9 | 5.1 | 10.6 |
| Vegetative Stage, 6 forage-crop average¶ | 20.2 | 3.3 | 16.7 | 51.9 | 0.32 |
| Milk Stage, 6 forage-crop average¶ | 21.3 | 13.1 | 32.3 | 26.5 | 1.22 |
| Soft Dough Stage, 6 forage-crop average¶ | 20.7 | 18.8 | 46.3 | 8.3 | 5.58 |
| * (Harstad, Steinshamn, 2010) |  |  |  |  |  |
| † (Lock, Bauman, 2004) |  |  |  |  |  |
| ‡ (Duvick et al., 2006), Table 2, commercial corn belt hybrid #1. | |  |  |  |  |
| § (Darby et al., 2013), Table 7 |  |  |  |  |  |
| ║(Darby et al., 2012), Table 7 |  |  |  |  |  |
| ¶ (Darby et al., 2012) (forage oats, barley, wheat, triticale, spelt, forage turnip) | | |  |  |  |
