# Supplementary material for: Enhancing the fatty acid profile of milk through forage‐based rations, with nutrition modeling of diet outcomes
Source: Food Sci Nutr. 2018 Feb 28;6(3):681–700. doi: 10.1002/fsn3.610 (PMC5980250; doi:10.1002/fsn3.610)
Supplement: Supplementary file 4 [file FSN3-6-681-s004.docx]

**Supporting Information**

**Fatty acid composition of dairy rations**

The FA composition of dairy cow rations affects the FA profile of their milk. Table S2 compares the FAs in natural forages with those of several cereal crops at various stages of maturity. All forage feeds are high in ALA, with very low LA/ALA ratios between 0.26 and 0.54, whereas mature grains have high LA/ALA ratios ranging from 10.6 for barley to 49 for corn. For the immature cereals and mixtures shown in Table S2, the LA/ALA ratios are low at the pre-boot, vegetative stage (0.32 to 1.12), but increase to 1.22 about 1 month later at the “milk stage” and further to 5.6 at the more mature “dough stage.” Most grass-fed dairy and beef certification programs allow feeding of cereal crops only at the vegetative, pre-boot stage.
